# PRIGo: a new multi-axis goniometer for macromolecular crystallography

**DOI:** 10.1107/S1600577515005354

**Published:** 2015-05-09

**Authors:** Sandro Waltersperger, Vincent Olieric, Claude Pradervand, Wayne Glettig, Marco Salathe, Martin R. Fuchs, Adrian Curtin, Xiaoqiang Wang, Simon Ebner, Ezequiel Panepucci, Tobias Weinert, Clemens Schulze-Briese, Meitian Wang

**Affiliations:** a Swiss Light Source, Paul Scherrer Institute, Villigen PSI, Switzerland; b Centre Suisse d’Electronique et Microtechnique SA, Neuchâtel 2002, Switzerland; c Dectris Ltd, Baden 5400, Switzerland

**Keywords:** multi-axis goniometry, macromolecular crystallography, diffractometer, diffraction data, collection strategy, beamline endstation

## Abstract

The Parallel Robotics Inspired Goniometer (PRIGo) is a novel compact and high-precision goniometer providing an alternative to (mini-)kappa, traditional three-circle goniometers and Eulerian cradles used for sample reorientation in macromolecular crystallography. Based on a combination of serial and parallel kinematics, PRIGo emulates an arc. It is mounted on an air-bearing stage for rotation around ω and consists of four linear positioners working synchronously to achieve *x*, *y*, *z* translations and χ rotation (0–90°), followed by a ϕ stage (0–360°) for rotation around the sample holder axis. Owing to the use of piezo linear positioners and active correction, PRIGo features spheres of confusion of <1 µm, <7 µm and <10 µm for ω, χ and ϕ, respectively, and is therefore very well suited for micro-crystallography. PRIGo enables optimal strategies for both native and experimental phasing crystallographic data collection. Herein, PRIGo hardware and software, its calibration, as well as applications in macromolecular crystallography are described.

## Abstract 2

The design and performance of the new multi-axis goniometer PRIGo developed at the Swiss Light Source at Paul Scherrer Institute is described.

## 1. Introduction   

The success of a macromolecular crystallography (MX) diffraction experiment depends on the accurate measurement of intensities and the completeness of the data. The main causes of failure are overlapping reflections (Dauter, 1999 ▸), radiation damage (Blake & Phillips, 1962 ▸; Ravelli & Garman, 2006 ▸; Garman & Weik, 2011 ▸) and noise originating from various sources (Holton & Frankel, 2010 ▸). Consideration and optimization of these parameters are crucial for obtaining the best outcome (Dauter, 2005 ▸, 2010 ▸). To this end, crystallographers have always employed data collection protocols involving crystal alignment using multi-axis goniometers. They are still routinely used on X-ray laboratory sources to obtain complete data sets at high resolution from small molecule crystals (Helliwell, 1992 ▸). At synchrotron MX beamlines, however, multi-axis goniometers were almost abandoned after the arrival of two-dimensional detectors, which restricted motions around the sample, due to the risk of collision. In addition, these goniometers never met the specifications imposed by the trend towards collecting on ever smaller crystals with ever smaller X-ray beam sizes. Their presence at third-generation synchrotrons was thus limited to few beamlines (Rosenbaum *et al.*, 2006 ▸; Shi *et al.*, 2006 ▸; Ascone *et al.*, 2007 ▸) until collision issues were addressed with the development of an inverse mini-kappa goniometer head with dedicated control software at ESRF/EMBL Grenoble (Brockhauser *et al.*, 2011 ▸). Several such systems have been installed at synchrotron beamlines (Brockhauser *et al.*, 2013 ▸) but the vast majority of data sets remain collected from a single axis, primarily around ω.

Developed at the Swiss Light Source (SLS) at Paul Scherrer Institute, the Parallel Robotics Inspired Goniometer (PRIGo) (Glettig *et al.*, 2009 ▸, 2011 ▸) is a new type of multi-axis goniometer with high-end features such as micrometer precision around the main rotation axis ω, a large collision-free angular range, reduced self-shadowing and low-maintenance device mechanics. Fully integrated into the beamline software architecture, it enables the realisation of advanced data collection protocols for both native and experimental phasing experiments in a user-friendly manner. Herein we present the geometric model, calibration and accuracy of the PRIGo goniometer as well as its integration at beamline X06DA (PXIII)^1^ at SLS. New opportunities for anomalous diffraction data collection are also presented.

## 2. Hardware and software description of PRIGo   

**Figure 1:**
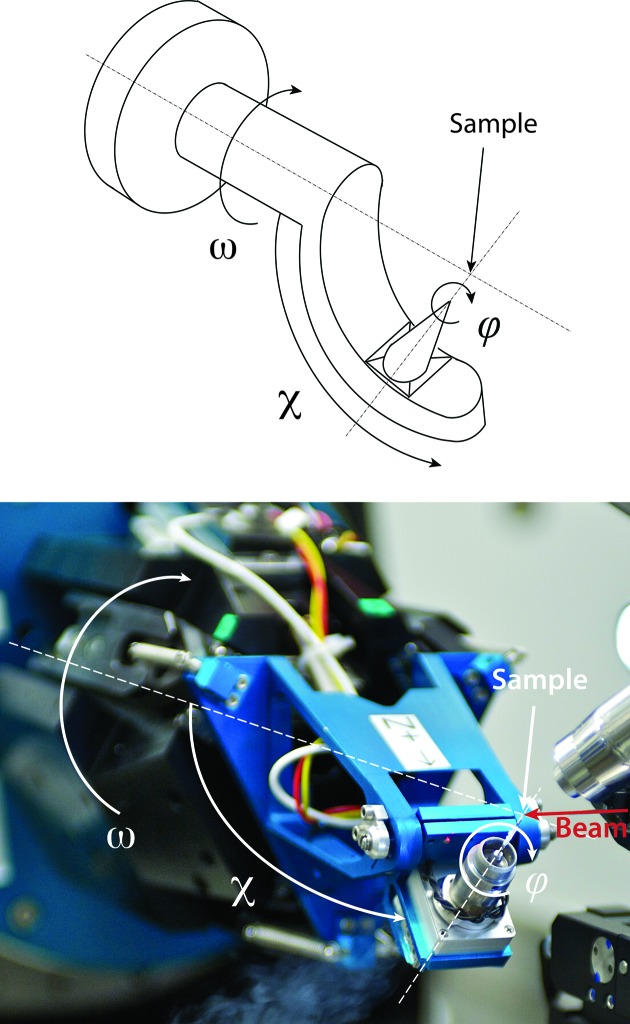
The PRIGo goniometer as mounted at beamline X06DA (PXIII) at SLS. The sketch of an arc (top) and a photograph of PRIGo at beamline X06DA (PXIII) at SLS (bottom) are represented with the ω, χ and ϕ angles overlaid. The beamline coordinate system is as follows: the *X* axis is along the spindle or ω axis, the *Y* axis is oriented vertically upward, and the *Z* axis is along the beam direction.

**Figure 2:**
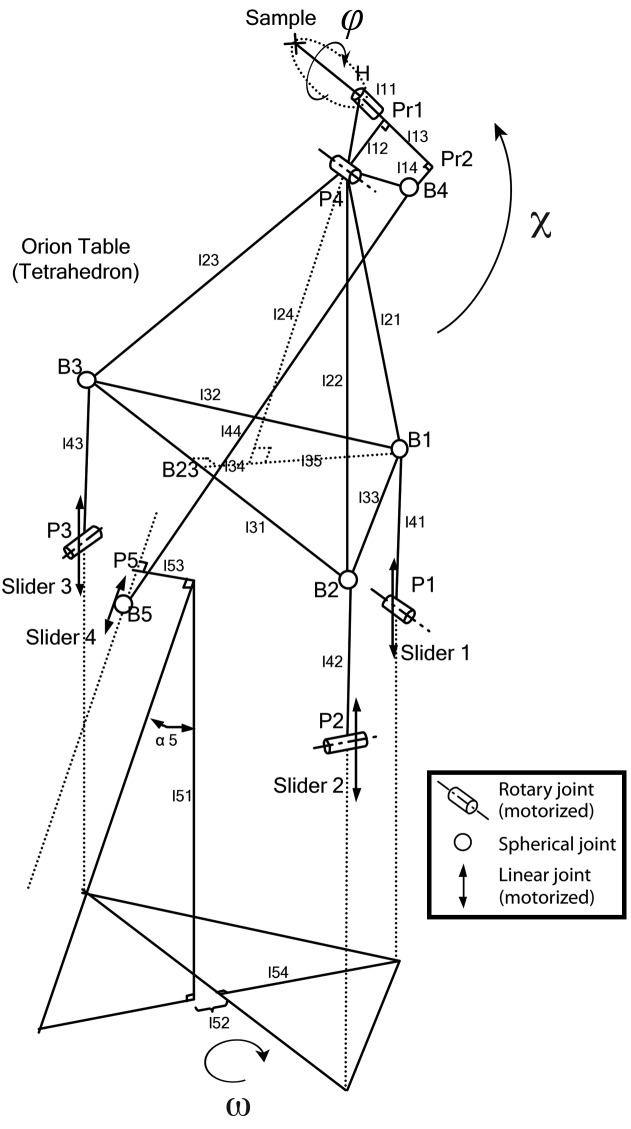
Kinematic structure of PRIGo. The PRIGo mechanism is driven by four linear positioners (or sliders), three of which are connected *via* a leg, comprising a pivot hinge and a ball joint, to a central tetrahedron shaped part, the so-called ‘Orion table’. The fourth linear positioner is responsible for tilting the ϕ rotation stage. Translations at the sample position, as well as the χ rotation, are achieved with synchronous movements of all four linear positioners. The ϕ stage rotates the sample around the sample holder axis, whereas the ω rotation can be performed by any conventional rotary stage. Numbered PRIGo parameters are preceded by l.

Based on a combination of serial and parallel kinematics, PRIGo is a compact and precise multi-axis goniometer, which emulates the movements of an arc (Fig. 1 ▸). PRIGo utilizes linear and rotary piezo positioners (SLC-P-2490, SLC-P-24120 and SR-P-2013-S, SmarAct GmbH) driven by a combined stick slip and direct current principle, with built-in linear encoders operating in closed loop and enabling nanometer resolution and positional reproducibility <10 nm. Three of the four linear positioners are parallel to the ω rotation axis and connected by spherical joints to a tetrahedron-shaped platform, called the ‘Orion table’ (Henein, 2000 ▸) (Fig. 2 ▸). The fourth positioner is inclined by an angle of 18° and connects a swing head supporting the ϕ rotational stage. PRIGo is mounted on an air-bearing rotary stage (Aerotech ABRS-250) used for the ω rotation with a 36-channel slip-ring in the center enabling unlimited rotary motion. Translations at the sample position, as well as the χ rotation, are achieved with synchronous movements of the four linear positioners.

**Figure 3:**
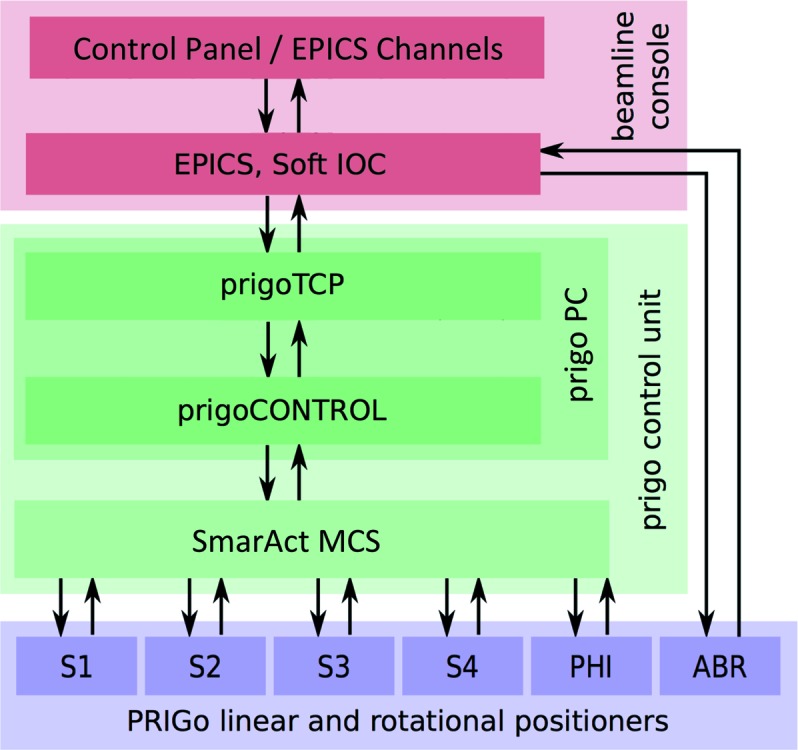
PRIGo software. The schematic shows the different modules of PRIGo’s software and their interactions (represented by arrows). PRIGo’s control system receives commands from the input/output control servers (IOC) of the EPICS framework, which also controls the other beamline components including the air-bearing rotary stage (ABR). Within the PRIGo control unit, a transmission control protocol (TCP) server handles all communication (commands and feedback) over a TCP socket connection. The TCP server also receives the ABR ω position *via* EPICS, which is used by the PRIGo control to perform the active correction (see §3). The SmarAct positioners (S1–S4: SmarAct positioners, PHI: SmarAct rotary stage) feedback loop is controlled by the modular control system (MCS) from SmarAct GmbH.

**Figure 4:**
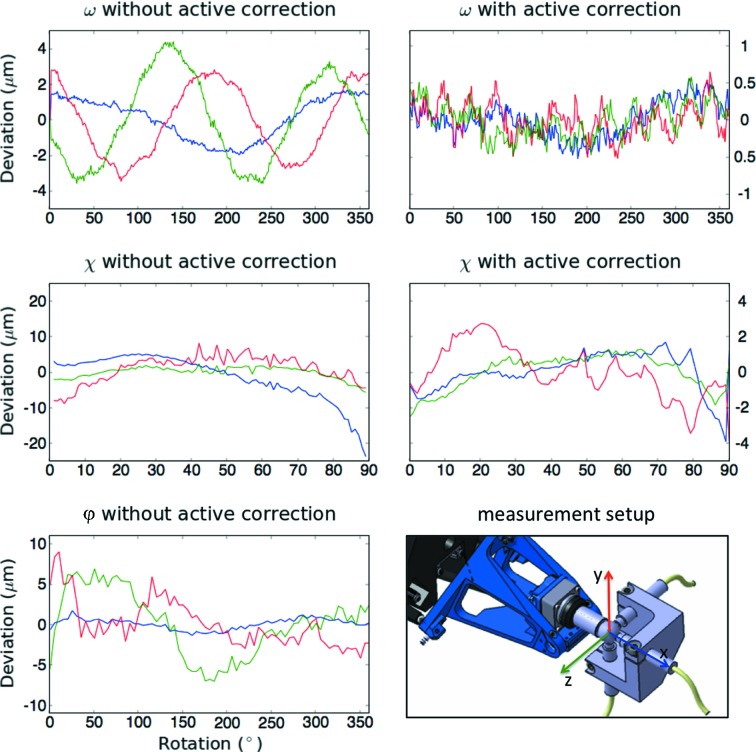
PRIGo’s sphere of confusion measurements for ω, χ and ϕ rotation axes. PRIGo SoC measurements and calibration were performed with three capacitive sensors aligned with the beamline coordinate system (see ‘measurement setup’ insert) and a metal sphere representing the sample. The plots represent the SoC obtained by optimization of PRIGo’s geometric model (without active correction) and after the implementation of an active correction (with active correction). The deviations along *x*, *y* and *z* from the ideal sample position are indicated in blue, red and green, respectively. During the measurement of an axis, the other two were kept at an angle of 0°. There is currently no active correction implemented for the ϕ axis. All SoC measurements were performed at 1° s^−1^ and reproducible within 200–300 nm.

PRIGo’s control system was implemented in the Orchestra Control Engine^2^, an open-source real-time control framework running on RTAI/Linux, which provides powerful and stable performance in a low-cost package. The various software modules of PRIGo (Fig. 3 ▸) communicate with a simple and efficient set of commands, keeping the network overhead to a minimum. PRIGo’s movements are described in the beamline coordinate system (see insert ‘measurement setup’ on Fig. 4 ▸), which does not correspond to linear movements of the individual motors. The conversion between the beamline coordinates (*x*, *y*, *z*, ω, χ, ϕ) and the motor positions (four linear positioners, ω and ϕ rotation stages) is achieved by a forward and inverse kinematics calculation. Performed by the control system at every cycle of the control loop (every 1 ms), this calculation ensures synchronized and smooth movements of the linear positioners.

## 3. Calibration of PRIGo   

The geometric model of PRIGo has 21 independents parameters, which correspond to PRIGo’s real dimensions (Fig. 2 ▸). Most could be measured using a three-dimensional coordinate-measuring machine (Wenzel LKS) with a precision of ∼10 µm, yielding the relative position of the four linear positioners (positions B1 to B5 on Fig. 2 ▸). The parameters linked to the fourth linear positioner (slider 4 on Fig. 2 ▸), which have the largest effect on the geometric model, were then refined in an iterative manner by minimizing the sphere of confusion (SoC) for the χ rotation axis (0–90°). Such SoC measurements were performed using a precise stainless steel reference sphere (Ø = 12.7 mm) with form tolerance below 0.25 µm, representing the sample mounted on the PRIGo sample holder and three capacitive sensors (Probe Type: C23/B, Driver System: Elite Series CPL190, Lion Precision/IBS Precision Engineering) aligned with the beamline coordinate system (Fuchs *et al.*, 2014 ▸) (Fig. 4 ▸). The same SoC measurements were also performed for the ω (0–360°) and ϕ (0–360°) rotation axes. We obtained a ‘geometric model’ peak-to-peak SoC of <5 µm, 25 µm and 10 µm for ω, χ and ϕ, respectively (Fig. 4 ▸, left side). To further improve these results, we implemented an active correction of the four motor positioners for the rotations around the ω (when χ = 0°) and χ axes (no correction was implemented for the ϕ axis yet). The active correction consists of parameterizing the measured deviation for a rotation (here, ω and χ), *i.e.* fitting a function to the SoC measurements. The deviations for the ω and χ rotations are described by a sum of two sinusoids (six parameters) and by a quadratic polynomial (five parameters), respectively. Implemented in the PRIGo control system, the active correction around the ω axis is applied up to a speed of 180° s^−1^. To achieve this, it is necessary to interpolate the position of the air-bearing rotary stage (ABR) ω position, which is obtained *via* EPICS (Fig. 3 ▸) at a rate of 10 Hz only. This is done within the calibration module of the PRIGo control, knowing the last ABR ω position and the rotation speed. The active correction is applied around the χ axis up to a speed of 5° s^−1^.

Owing to the high resolution and repeatability of the SmarAct positioners as well as the reproducibility of PRIGo’s mechanics, the SoC improved to <1 µm for ω and <7 µm for χ (Fig. 4 ▸, right side). This accuracy is valid within ±2.5 mm deviation along *x*, *y* and *z* from the ideal sample position. The calibration, together with the active correction, have proven to be extremely stable over time and were repeated only once after 15 months of operation, following the maintenance of two linear positioners.

## 4. Integration of PRIGo at beamline X06DA (PXIII) at SLS   

**Figure 5:**
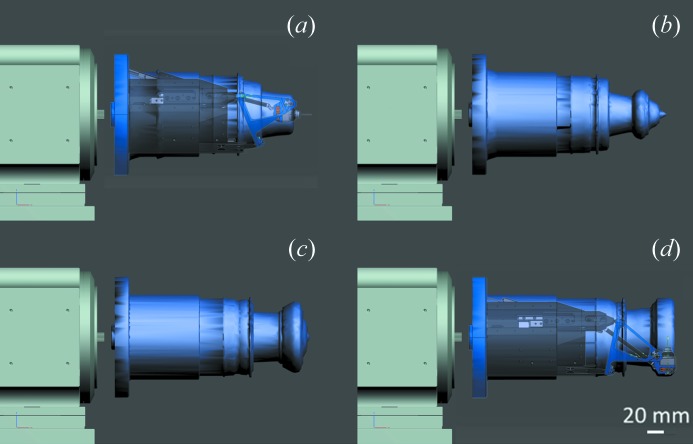
PRIGo space envelopes for full ω turns with different χ-offsets. Envelopes are shown for increasing χ values of (*a*) 0°, (*b*) 30°, (*c*) 60° and (*d*) 90. A view of PRIGo with a sample at the tip (standard 18 mm pin) is overlaid for (*a*) and (*d*) for better visualization of the goniometer’s parts.

**Figure 6:**
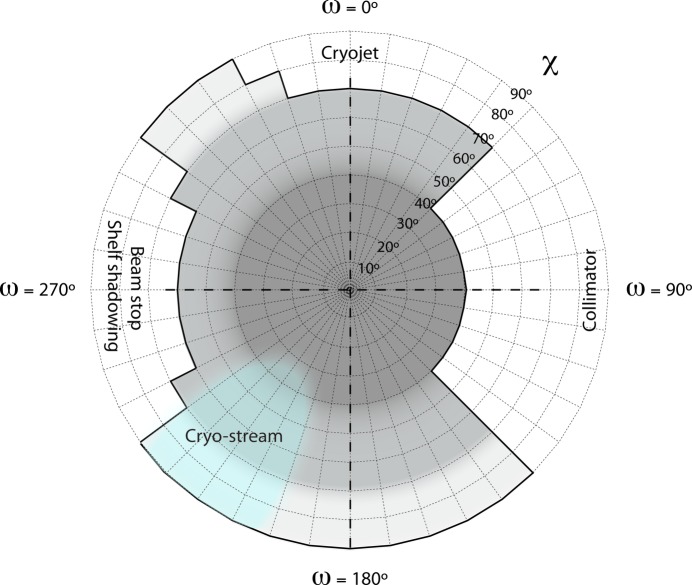
PRIGo collision and shadowing map at beamline X06DA (PXIII). Allowed χ angles with respect to the ω axis are shown in grey. 360° data can be collected around ω with a χ angle up to 40° (dark grey), more than 210° with a χ angle between 40° and 70° (medium grey), and more than 120° with a χ angle of 90° (light grey), respectively. Beamline devices with potential collision are indicated together with the position of the cryo-stream (light blue).

PRIGo was installed at beamline X06DA (PXIII) at the Swiss Light Source in January 2012 and is now fully integrated into the new data acquisition user interface DA+ (E. Panepucci *et al.*, unpublished). Its compact design and small space envelope at large χ angles (Maag *et al.*, 2013 ▸) (Fig. 5 ▸) maximize the achievable angular range but self-shadowing on the detector and potential collisions with beamline devices such as the collimator or the beam stop still impose angular restrictions. Currently, PRIGo offers collision-free ω rotation with a χ angle up to 40°, more than 210° ω rotation with a χ angle between 40° and 60°, and more than 120° ω rotation with a χ angle of 90° (Fig. 6 ▸). The cryogenic nitrogen gas blowing directly on parts of PRIGo (mainly the head) can result in deflection of the device and movement at the sample position (∼10 µm). This is not of practical concern at beamline X06DA (PXIII) because the cryojet is oriented such that the affected positions are limited (light blue on Fig. 6 ▸), and in the cryo-stream for only a short time (∼20 s) in a typical 360° data collection at a speed of 2° s^−1^.

In addition, the PRIGo ϕ axis can also serve as a data collection axis (Table 1 ▸) thanks to continuous shutterless data acquisition capabilities offered by fast readout detectors such as the PILATUS (Broennimann *et al.*, 2006 ▸; Henrich *et al.*, 2009 ▸). Finally, PRIGo is compatible with both sample changer usage [an IRELEC CATS system with vial mounting is in place at X06DA (PXIII)] and manual transfer where a χ offset can facilitate both crystal mounting and recovery. Possible bump or collision on PRIGo upon sample exchange (from either robot or manual mounting) are recovered with an initialization procedure, which brings back all linear positioners to their reference positions. This procedure is completed in about 10 s.

## 5. Applications   

Multi-axis goniometers like PRIGo are beneficial in a wide variety of cases including a simple reorientation to rotate around the crystal’s best spot or data collection while avoiding contamination from satellite crystals. Other advantages are the unambiguous space group determination, alignment of a long crystallographic axis along the spindle in order to avoid overlapping reflections or minimization of the total oscillation range needed for a complete data set on a radiation-sensitive crystal (Brockhauser *et al.*, 2013 ▸). For crystals diffracting to atomic resolution, data measured at a different χ angle to collect the ‘missing cusp’ yield complete data in the highest-resolution shell after merging.

**Figure 7:**
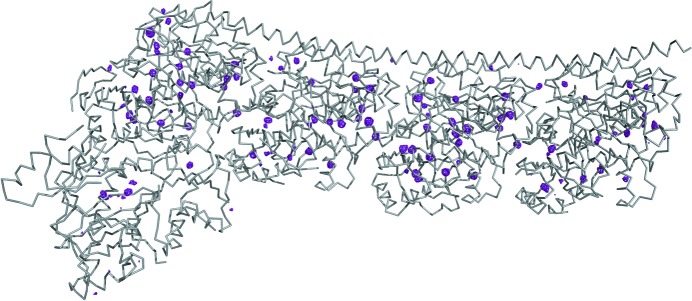
Cα-trace of the T_2_R-TTL complex (Prota *et al.*, 2013 ▸) and the anomalous difference Fourier map at 5σ. The anomalous difference map (magenta) shows the presence of 118 sulfur, 13 phosphorus, 3 calcium sites, 2 chloride sites in the multiprotein–ligand complex.

Multi-axis goniometers also enable optimal strategies for the measurements of anomalous diffraction data. In the case of single-wavelength anomalous dispersion (SAD) requiring high multiplicity (Dauter *et al.*, 1999 ▸), they provide an elegant way to acquire true redundant data (Debreczeni *et al.*, 2003 ▸) with improved accuracy by changing the crystal orientation (here, multiple low-dose 360° data sets at various χ and ϕ angles). We successfully implemented such a strategy at beamline X06DA (PXIII) at SLS (Weinert *et al.*, 2015 ▸) and solved, using only the anomalous signal of low-*Z* elements and a single crystal entity, a 266 kDa multiprotein–ligand tubulin complex (Prota *et al.*, 2013 ▸) (T_2_R-TTL) (Fig. 7 ▸). With 2317 residues, this is the largest structure solved by native-SAD phasing to date.

In the case of multiple-wavelength anomalous dispersion (MAD) experiments, PRIGo can be used to align an even-fold symmetry axis parallel to the spindle in order to collect *Bijvoet* mates on the same image, *i.e.* with essentially similar X-ray dose and absorption effects (Kahn *et al.*, 1985 ▸; Hendrickson *et al.*, 1988 ▸). For this, we use *XOalign* (Legrand, 2009 ▸) with an orientation matrix from indexed diffraction patterns to calculate possible goniometer angles compatible with the beamline collision map (Fig. 6 ▸). Phasing experiments can also be designed to take advantage of the anisotropy of the anomalous signal at an absorption edge (Bricogne *et al.*, 2005 ▸; Schiltz & Bricogne, 2008 ▸, 2010 ▸). Here, the possibility offered by PRIGo to collect data around the ϕ axis and to orient this axis at a chosen angle with respect to the X-ray beam polarization is potentially a great advantage.

In conclusion, with a large angular range and micrometer precision, we have shown that the multi-axis goniometer PRIGo offers new possibilities for optimizing MX data collection protocols. By facilitating multi-axis data acquisitions, we aim at encouraging crystallographers to measure data with the highest accuracy, especially when attempting to obtain phases experimentally.

## Figures and Tables

**Table 1:** Data collection and processing statistics of a lysozyme crystal collected with PRIGo at beamline X06DA (PXIII) Data sets of 360 were collected on a PILATUS 2M-F detector from a single lysozyme crystal in three different ways: around the axis (‘traditional’ rotation axis), around the axis with a angle of 0 (*i.e.* similar to the data collection around ), and around the axis with a angle of 90 (simulating a vertical axis). The three data sets were collected in an interleaving manner with 90 wedges in order to distribute the X-ray dose evenly. Still, small effects of radiation damage can be observed in the processing statistics in the last resolution shell. An expected lower completeness was observed for the ‘vertical axis’ data as a result of both Lorentz and polarization corrections.

Data sets	Around	Around with = 0	Around with = 90 (‘vertical axis’)
Space group	*P*4_3_2_1_2		
Unit cell	*a* = 78.3, *b* = 78.3, *c* = 37.2		
Beamline	X06DA (PXIII) / SLS		
Wavelength ()	1.0		
Overall / inner / outer resolution shells ()	39.11.2 / 39.110.0 / 1.31.2		
Completeness (%)	99.9 / 99.8 / 99.6	99.9 / 99.8 / 99.1	97.9 / 99.8 / 91.2
*R*_meas_ (%)	3.3 / 2.0 / 87.5	3.5 / 2.1 / 96.1	3.3 / 2.1 / 101.7
*I*/(*I*)	46.1 / 151.3 / 3.6	43.8 / 144.9 / 3.2	42.9 / 140.7 / 2.6
CC(1/2)	100.0 / 100.0 / 89.3	100.0 / 100.0 / 87.0	100.0 / 100.0 / 79.7
Reflections: measured	875773	877808	725366
unique	36675	36680	35958

## Footnotes

1 X06DA (PXIII), http://www.psi.ch/sls/pxiii/.

2 Orchestra, http://en.wikipedia.org/wiki/Orchestra_Control_Engine.
